# A clinical decision rule for the use of plain radiography in children after acute wrist injury: development and external validation of the Amsterdam Pediatric Wrist Rules

**DOI:** 10.1007/s00247-015-3436-3

**Published:** 2015-08-23

**Authors:** Annelie Slaar, Monique M. J. Walenkamp, Abdelali Bentohami, Mario Maas, Rick R. van Rijn, Ewout W. Steyerberg, L. Cara Jager, Nico L. Sosef, Romuald van Velde, Jan M. Ultee, J. Carel Goslings, Niels W. L. Schep

**Affiliations:** Department of Radiology, Academic Medical Centre, University of Amsterdam, Meibergdreef 9, 1105, AZ Amsterdam, The Netherlands; Trauma Unit, Department of Surgery, Academic Medical Centre, University of Amsterdam, Amsterdam, The Netherlands; Emergency Department, Academic Medical Centre, University of Amsterdam, Amsterdam, The Netherlands; Department of Public Health, Erasmus MC - University Medical Centre, Rotterdam, The Netherlands; Department of Surgery, Spaarne Hospital, Hoofddorp, The Netherlands; Department of Surgery, Tergooi Hospitals, Hilversum, The Netherlands; Department of Surgery, Sint Lucas Andreas Hospital, Amsterdam, The Netherlands; Department of Surgery, Maasstadziekenhuis Rotterdam, Rotterdam, The Netherlands

**Keywords:** Child, Clinical decision rule, Distal forearm fracture, Multicenter prospective study design, Radiography, Trauma, Wrist

## Abstract

**Background:**

In most hospitals, children with acute wrist trauma are routinely referred for radiography.

**Objective:**

To develop and validate a clinical decision rule to decide whether radiography in children with wrist trauma is required.

**Materials and methods:**

We prospectively developed and validated a clinical decision rule in two study populations. All children who presented in the emergency department of four hospitals with pain following wrist trauma were included and evaluated for 18 clinical variables. The outcome was a wrist fracture diagnosed by plain radiography.

**Results:**

Included in the study were 787 children. The prediction model consisted of six variables: age, swelling of the distal radius, visible deformation, distal radius tender to palpation, anatomical snuffbox tender to palpation, and painful or abnormal supination. The model showed an area under the receiver operator characteristics curve of 0.79 (95% CI: 0.76-0.83). The sensitivity and specificity were 95.9% and 37.3%, respectively. The use of this model would have resulted in a 22% absolute reduction of radiographic examinations. In a validation study, 7/170 fractures (4.1%, 95% CI: 1.7-8.3%) would have been missed using the decision model.

**Conclusion:**

The decision model may be a valuable tool to decide whether radiography in children after wrist trauma is required.

## Introduction

In children, wrist trauma is one of the most common reasons for visiting the emergency department [1–3]. A fracture of the wrist accounts for approximately 25-36% of all pediatric fractures [4–8]. The most common diagnosed type of injury following wrist trauma is a fracture of the distal forearm. Occurrence of carpal fractures is low, varying from 1% to 3% in children with a wrist fracture [7–9].

During the last 4 decades, an increase of distal forearm fractures in children was reported [2, 3, 6]. Due to the increase in incidences, health care costs for pediatric forearm fractures in the United States currently exceed $2 billion per year [10]. An important cause for this rise in health care costs is the increase in the number of radiographs requested [3, 11].

Unlike ankle and cervical spine injury [12–14], no guidelines are available that indicate when children with wrist trauma require radiography. Therefore, radiographic imaging in children following acute wrist trauma is often performed routinely in most hospitals [15, 16]. However, in one study only 51% of radiograph studies performed in children after wrist trauma demonstrated a fracture [17].

Because of this routine referral for radiography, unnecessary costs are incurred, waiting time is extended and radiation exposure is increased [18–21].

The goal of this study was twofold: 1) to derive a clinical decision tool, and 2) to externally validate a clinical decision tool that physicians can use to decide whether referral for radiography in children with acute wrist trauma is required and consequently whether this would lead to a reduction in the number of radiographs requested.

## Materials and methods

### Design and setting

This study was part of a combined study in which the adult population was analysed separately from the pediatric population. The study protocol of the adult patient group has previously been published [22]. In the pediatric population, we applied practically the same protocol. The results are addressed in this article. We performed a multicenter prospective study from April 6, 2011, to April 15, 2014, in four national hospitals -- one university hospital and three non-university teaching hospitals. The children included in the university hospital formed the development cohort. The children included in the three other hospitals formed the validation cohort. We did not expect a difference in referral patterns among hospitals since the university hospital also functions as a local referral center for general practitioners.

The study consisted of three components: 1) to prospectively define a clinical decision tool, 2) to externally validate this clinical decision tool and (3) to define a clinical decision tool.

The Medical Ethical Review Committees of all participating hospitals approved the study (Dutch Trial Registry number NTR2651) and waived informed consent.

### Participants

All children younger than 18 years old who presented in the emergency department in one of the four participating hospitals with pain following wrist trauma were included. Children younger than 3 years old were excluded, as it is difficult to obtain an objective physical examination. We also excluded patients whose injury occurred more than 72 h previously or patients who had sustained multiple injuries (Injury Severity Score ≥16). Patients whose radiographs were requested previous to their visit to the emergency department (e.g., by their general practitioner) were excluded as well [22]. Patients with pre-existing musculoskeletal disease, coagulopathy or developmental delay and patients with previous history of surgery or recent (<3 months) injury of the affected wrist were also excluded. Physicians were instructed not to include patients if they were aware of the outcome of the radiograph performed before physical examination. Since it was not mandatory to obtain radiography in all children following wrist trauma only 12 out of 897 patients (1.3%) did not undergo radiographic imaging. These children were also not included in the study.

### Definitions

Wrist trauma was defined as any high or low energetic accident involving the wrist. Corresponding to the protocol of the adult study population, wrist injury was defined as injury to the proximal segment of the hand, including the carpal bones and the associated soft parts, and the distal one third of the ulnar and radial bone [22]. Since the incidence of carpal fractures in children is low and since scaphoid fractures are frequently occult on plain radiography, carpal fractures were not taken into account [7, 9, 23]. A fracture was defined as a disruption of one or more of the cortices of the bone. Buckle fractures or bowing fractures were also recorded as a true fracture, as were fissures and avulsions. A combined fracture of the ulna and radius, known as an antebrachii fracture, was recorded as one fracture.

### Data collection and variables

We used standardized case record forms to prospectively collect our data in all four participating hospitals. The case record form consisted of 18 clinical variables, including patient characteristics, physical examination and functional testing (Appendix 1). All variables were selected after evaluation of previous studies and consensus agreement of clinical experts [24–26]. All variables on the case record form, in exception of grip strength, were dichotomous (yes/no). The attending physician included eligible children after physical examination. The case record forms were filled in after physical examination. The assessors were all physicians and included consultant emergency medicine physicians, general practice registrars, and specialist registrars of the departments of (trauma) surgery, emergency medicine or orthopedics. All physicians received regular instructions and training before recruiting children to the study. Additionally, informative pocket cards and posters were provided. In order not to disrupt common practice, referral for radiography was left to the discretion of the attending physician.

### Test methods

The outcome was the presence or absence of a radiologically detected fracture of the distal forearm (radius, ulna or both) diagnosed by the attending radiologist. A third-year resident in radiology (A.S.) and a clinical physician (M.M.J.W.) revised all radiographic imaging and radiologic reports. Any discrepancies in diagnosis were resolved in consensus reading. Where necessary, a pediatric radiologist (R.R.vR.) with more than 10 years of experience was consulted.

Regular clinical information was available for the radiologist, but the content of the case record form was not provided. Conforming to standard clinical practice, plain radiographic imaging consisted of at least one posterior-anterior and one lateral view and any further conventional imaging available (e.g., scaphoid series).

### Sample size

A common rule of thumb to determine the sample size of the development of a prediction model is 10 events per variable [27]. Since our case record form (CRF) consisted of 18 variables, the inclusion of a minimum of 180 children who sustained a fracture was required. External validation required at least 100 events (fractures) and 100 non-events [27].

### Statistical analysis

For efficient statistical analysis, we used imputation techniques to input the missing values (*aregImpute* function from the *Hmisc* library, R, version 3.0.1.) [28–30]. For each missing variable, this algorithm initializes the values from a random sample from the non-missing values. Using this data, it then fits a flexible model that predicts the missing target variable while finding its optimum transformation. Each missing value is imputed with the observed value whose predicted transformed value is closest to the predicted transformed value of the missing variable. We considered an imputation model that included all potential predictor variables and the outcome. The first set of imputations was used for the analyses.

#### Model development and internal validation

We derived a clinical prediction model from data on patients enrolled in the university hospital.

We fitted a logistic regression model with 18 predictors, which was reduced using a stepwise backward elimination process based on a *P*-value of 0.15 [31]. We used bootstrapping to estimate the internal validity (500 replications). Bootstrapping mimics the process of sampling from the underlying population and is a method to quantify the optimism of a prediction model: the difference between performance in the bootstrap sample and performance in the original sample [32]. A shrinkage factor, also obtained by bootstrap validation, was used for multiplication of the regression coefficients.

### External model validation and final model development

To assess general applicability, we validated the model in the cohort that included all children enrolled in the three other participating hospitals. For each patient in the three other hospitals (the validation cohort), the probability of a distal forearm fracture was calculated using the prediction model. To estimate the ability of the model to discriminate between patients with and without a fracture, we calculated the area under the receiver operating characteristics curve (AUC). An AUC of 0.5 means that the test is not predictive. An AUC of 1.0 means that the predictive value is very high. The agreement between observed outcomes and predictions (the calibration of the model) was determined by plotting the predicted probability of a fracture and the observed frequency of a fracture. A slope of 1 is ideal for the observed outcomes versus predicted risk [31].

In order to provide a recommendation (whether to perform radiography or not), we established a cutoff value for a predicted probability. Previous literature used a threshold varying from 20% to 25% for the use of radiography in children and adolescents for detecting upper extremity injury [33]. Therefore, we used a threshold probability of 23% (the mean of 20-25%), beyond which the Amsterdam Pediatric Wrist Rules recommend radiographic imaging for all children with wrist trauma and below which none would undergo radiographic imaging.

As a final step, the model was fitted on data from both cohorts combined to obtain the final estimates of the regression coefficients.

## Results

### Participants

**Fig. 1:**
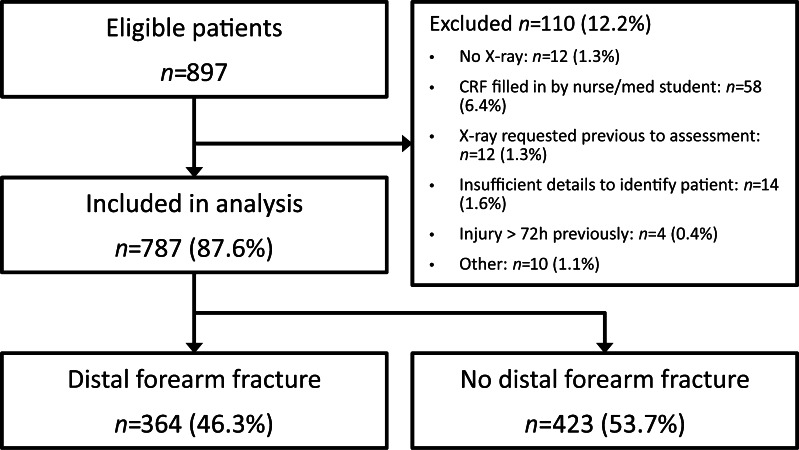
Flowchart demonstrates patient selection and outcomes

A consecutive series of 897 children with wrist injury was recruited in the four participating hospitals. We excluded 110 patients (12.2%) for various reasons (Fig. 1). In 364 children (46.3%), a fracture of the distal forearm was diagnosed (Table 1). In the development cohort (the university hospital), we included 408 patients. The mean age was 12 years (standard deviation: 3.0); more than half of them were male (66.7%). A fracture of the distal forearm was diagnosed in 194 patients (47.5%). In the validation cohort (three teaching hospitals), 379 patients were included. There were no significant differences between the cohorts (Table 1). The mean age in the validation cohort was 11 years (standard deviation: 2.9) and 53% were male. In 170 patients (44.9%), a fracture of the distal forearm was diagnosed. The observers had several months up to 21 years of experience in the emergency department (median: 3.5, interquartile range: 2–11).

**Table 1:** Clinical and demographic characteristics of the development cohort, validation cohort and total cohort

Characteristics	Development cohort^a^ (*n* = 408)	Validation cohort^b^ (*n* = 379)	Total^c^ (*n* = 787)
Median age, years (SD)	12 (3)	11 (2.9)	11 (2.9)
Males (%)	272 (66.7)	201 (53.0)	473 (60.1)
Patients with a fracture of the distal forearm (%)	194 (47.5)	170 (44.9)	364 (46.3)
Fractures	207	180	387
Distal radius	165 (79.8)	155 (86.1)	320 (82.7)
Distal ulna	2 (0.97)	0 (0.0)	2 (0.52)
Forearm	27 (13.0)	15 (8.3)	42 (10.9)
Other^d^	13 (6.3)	10 (5.6)	23 (5.9)

*SD* standard deviation

^a^Data from the academic hospital, the initial development cohort

^b^Data from the other three hospitals, the validation cohort

^c^Patients included in the analysis (data from all four hospitals), the final development cohort

^d^Fractures of the carpal bones and metacarpal bones

### Missing values and imputation

In both the development and validation cohorts, 83% of the cases were complete. Missing values comprised less than 5% for all variables with the exception of prehensile grip strength, which was not completed in 12.5% of the patients (Appendix 2).

#### Model development

The clinical prediction model derived included six variables: age, swelling of the distal radius, visible deformation, distal radius tender to palpation, anatomical snuffbox tender to palpation and painful supination (Table 2). The AUC of the model was 0.81 (95% CI: 0.76-0.85); after correction for optimism by bootstrapping the AUC was 0.77 (95% CI: 0.73-0.82). We evaluated lack of fit of the model by relaxing assumptions of linearity and additivity of predictor effects. We hereto examined nonlinear transformations of the variable age, including the square term and the log transformations. We also examined interaction terms between swelling of the distal radius and painful palpation, swelling of the distal radius and visible deformation and painful palpation (Appendix 3). We found no evidence of non-linearity of the effects of age and none of the interactions terms was statistically significant.

**Table 2:** Contribution of variables as predictors of the presence of a distal forearm fracture in the clinical decision rule

Predictor	Coefficient (95% confidence interval)
Age	−0.14 (−0.22 – −0.061)
Swelling of distal radius present	1.18 (0.706–1.65)
Visible deformation	1.58 (0.412–2.745)
Bone tenderness distal radius	1.14 (0.278–2.004)
Bone tenderness of anatomical snuff box	−1.75 (−2.37 – −1.136)
Painful supination	0.52 (0.006–1.028)

### External model validation and test characteristics

The external performance of the model was assessed in the 379 patients in the validation cohort. The AUC of the external validation was 0.79 (95% CI: 0.76-0.82) and the calibration slope 1.07 (95% CI: 0.82-1.33). After applying a threshold of 23%, the sensitivity and specificity of the Amsterdam Pediatric Wrist Rules for detecting fractures of the distal forearm in the validation cohort were respectively 95.9% (95% CI: 91.7-98.0%) and 37.3% (95% CI: 31.0-44.1%) (Table 3). The Amsterdam Pediatric Wrist Rules led to an absolute reduction of 22% of requested radiographs.

**Table 3:** Test characteristics and performance of the Amsterdam Pediatric Wrist Rules in the external validation cohort as tested on data from three hospitals (the validation cohort), cutoff point for radiograph yes or no was a predicted probability of fracture of 23%

	Patients with fracture	Patients without fracture	Total
Amsterdam Pediatric Wrist Rules indicate radiograph	163	131	294
Amsterdam Pediatric Wrist Rules indicate no radiograph	7	78	85
Total	170	209	379
Sensitivity (95% confidence interval)	95.9 (91.7–98.0)		
Specificity (95% confidence interval)	37.3 (31.0–44.1)		

After applying the Amsterdam Pediatric Wrist Rules, 7/170 fractures (4.1%, 95% CI: 1.7-8.3%) were missed in the external validation cohort (Appendix 4). They consisted of six buckle fractures of the distal radius and one non-displaced distal radius fracture with a buckle component. All these missed fractures were found in boys ages 10-15 years old.

## Discussion

Our derived prediction model, the Amsterdam Pediatric Wrist Rules, is a valuable tool for physicians in the emergency department in deciding if referral for radiography is required in children after acute wrist trauma. We showed that a combination of six clinical variables was able to discriminate between children with and without a fracture with an AUC of 0.79.

By applying the Amsterdam Pediatric Wrist Rules, the number of requested radiographs would have been reduced by 22%. The incidence of children with a fracture in the Netherlands in 2009 was 4.465 per 100,000 children from 5–19 year old [6]. Since approximately 50 % of the children with wrist injury are diagnosed with a fracture, this resulted in 8,930 children with wrist injury per 100,000 children in 2009 [17, 34]. By applying the Amsterdam Pediatric Wrist Rules, radiographic imaging could have been prevented in almost 2,000 children per 100,000 (22% reduction). At a price of 48 Euro/$50 per radiograph, the possible reduction of health care cost will be 96.000 euro per 100,000 children annually [16, 17]. This amount is probably an underestimation because the provided incidence included children ages 5–19 years old and the population that could benefit from the Amsterdam Pediatric Wrist Rules is 3–18 years old. As was the case following the implementation study of the Ottawa Ankle Rules, a reduction in waiting time may be expected after applying the Amsterdam Pediatric Wrist Rules [35]. Additionally, we assume that applying the Amsterdam Pediatric Wrist Rules will generate a reduction in radiation exposure. Although radiation exposure of plain radiography of the wrist is low (effective dose: 0.16 μSv), it is important to prevent unnecessary radiation exposure according to the ALARA principle (As Low As Reasonably Achievable), especially in children [11, 36]. Obtaining a US for detecting wrist fractures in children might also reduce radiation exposure; however, only a few studies have been performed, all with small study groups [36, 37]. Moreover, it is unclear if the use of sonography leads to a reduction in health care costs.

After applying the Amsterdam Pediatric Wrist Rules in seven patients (4.3%), a fracture would have been missed. The missed fractures consisted of six buckle fractures of the distal radius and one non-displaced distal radius fracture with a buckle component without displacement. According to literature and an expert panel consisting of two pediatric surgeons, one trauma surgeon and one orthopedic surgeon, none of these fractures needed closed reduction or operative treatment, but would have been treated with a splint [38–40]. This type of treatment is identical to treatment for children without a fracture who are diagnosed with a contusion or sprain of the wrist. We also expect that in children in a lot of pain, physicians are more likely to give a cast for pain regulation. Therefore, we consider that the treatment and prognosis would not have been influenced by a missed or delayed diagnosis [41]. Moreover, in children with stable buckle fractures, it is known that subacute treatment does no lead to adverse clinical outcomes [42]. However, a follow-up evaluation by telephone, or the advice to contact the hospital if symptoms remain after 1 week, can be considered for patients who did not initially require a radiograph, according to the Amsterdam Pediatric Wrist Rules.

Because physicians were not obligated to refer patients for radiography, in 12 patients no radiograph of the wrist was obtained. These patients were not included in the study, but none of these 12 children returned to the hospital for persisting complaints in the following 4 weeks.

A limitation of the Amsterdam Pediatric Wrist Rules is its specificity of 37.3%. We could have generated a higher specificity by using another threshold, but this would have led to a decrease in the sensitivity and thus an increase of missed fractures. In accordance with Maguire et al. [43], we judged it would not be applicable since it misses >5% of fractures in children. Since we aimed to reduce the number of requested wrist radiographs, a threshold compromise between missed fractures and reduction of radiographs was chosen [33, 44]. According to the literature, we determined that about three avoided radiographs outweigh one missed fracture and therefore we used a threshold value of 23.0% (1/25) for the predicted probability [33]. The sensitivity prediction rule was 96%. Adding anamnestic variables to the model could possibly strengthen our prediction rule and result in a higher sensitivity. However, since children are not always capable or trustworthy of telling what type of trauma occurred, we did not take clinical history variables into account.

Another limitation is that some patients with wrist pain were missed due to crowding in the emergency department. This might have introduced a selection bias. However, we expect that the reasons for missing patients were mostly related to emergency department crowding and not to patient characteristic. Therefore, we consider this bias minimal.

We might have introduced another type of selection bias since this study took place in only university hospitals and non-university teaching hospitals, and not in a non-teaching hospital. We assume that in non-teaching hospitals the referral for radiography is routinely done by triage nurses, while in (university) teaching hospitals the referral for radiography is usually done by physicians. Upcoming studies should reveal if the Amsterdam Pediatric Wrist Rules could also be applied by triage nurses. Nevertheless, we expect that the clinical signs and the incidence of wrist fractures in children in non-teaching hospitals do not significantly differ from (university) teaching hospitals and therefore we do not expect that this has significantly influenced our results.

The final limitation of our study is that in 12.5% of the CRFs the valuable prehensile grip strength was not completed. In several cases, the physician wrote that this was because the patient was in too much pain to perform this test. However, the difference between patients with and without prehensile grip strength as a missing variable was small and therefore it is not likely that our results were influenced by the imputation of this variable.

Three preceding studies have considered the diagnostic value of physical findings in children with acute wrist trauma. In 1986, Rivara et al. [26] retrospectively studied 116 children and found gross deformity and point tenderness to be the best predictors for a fracture of the upper extremity, with a sensitivity of 81% and a specificity of 82%. The sample size and, more importantly, the sensitivity of this study were much lower than in our study results. In 2000, Pershad et al. [24] performed a prospective study in 48 children and found that a 20% or more reduction of grip strength and distal radius point tenderness were predictive values for the presence of a wrist fracture. These clinical predictors had a sensitivity of 79% and a specificity of 63%. However, this study was also limited by a small sample size.

A study performed in 2006 in 227 children showed that radial tenderness, focal swelling and abnormal supination/pronation were associated with wrist fractures in children [25]. These predictive variables showed a sensitivity of 99.1% and specificity of 24%. The predictive variables and sensitivity of these variables were almost similar, but our specificity was higher and thus the potential reduction of the amount of requested radiographs in our study is higher (22% vs. 13%).

None of the decision rules is externally validated, which is recommended [45]. The Amsterdam Pediatric Wrist Rules did undergo external validation in a study population with different type of hospitals and physicians.

**Fig. 2:**
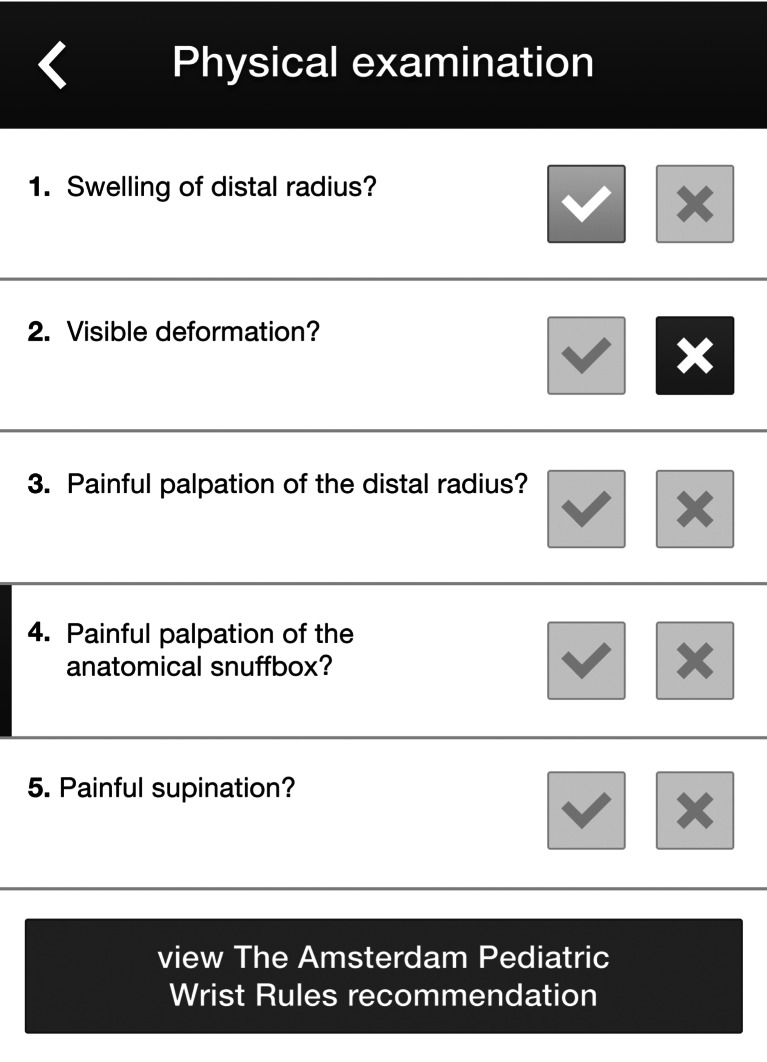
Screenshot of the smartphone application of the decision model used for the implementation study (built by ©Applicationbuilders, Amstelveen, The Netherlands)

An upcoming implementation study will evaluate the impact of the Amsterdam Pediatric Wrist Rules on the number of radiographs, emergency department waiting times and health care costs. The formula to predict the probability of a fracture (Table 4) will be made available in a smartphone application (Fig. 2). This application will give physicians a recommendation if radiography is recommended according to the probability of a distal forearm fracture.

**Table 4:** Linear predictors and probability. Coefficients were derived from a fit of the model on both cohorts combined (*n* = 787). All individual parameters add to the probability of a fracture

Linear predictor:
−0.185 x age (years) + 1.144 (if swelling of distal radius present) + 1.56 (if visible deformation present) + 1.183 (if bone tenderness of distal radius present) -1.424 (if bone tenderness of anatomical snuff box present) + 0.356 (if supination painful) + 0.466
Probability of a fracture based on final model:
1/ (1 + EXP (−Linear predictor))

## Conclusion

The derived clinical decision model (Amsterdam Pediatric Wrist Rules) may be used as a tool for physicians in the emergency department in deciding if referral for radiography in children after acute wrist trauma is necessary. Applying the model, 7/170 fractures (4.1%, 95% CI: 1.7-8.3%) were missed in an external validation study.

## Appendix 1

**Table:** Clinical variables recorded

Sex
Age
Swelling of distal radius
Swelling of distal ulna
Swelling of anatomical snuffbox
Visible deformation
Bone tenderness
Distal radius
Distal ulna
Anatomical snuffbox
Active mobility painful^a^
Dorsiflexion
Palmar flexion
Supination
Pronation
Ulnar deviation
Radial deviation
Functional tests painful^a^
Radio ulnar ballottement test^b^
Axial compression of forearm
Prehensile grip strength^c^

^a^Items were scored positive if the patient experienced pain, if they were unable to perform the test or if they refused to perform the test

^b^Test is positive if pain or tenderness occurs when the ulna is translated from volar to dorsal while the radius manually fixated

^c^Both sides assessed three times with a Baseline Hydraulic Hand Dynamometer, expressed in percentage of decrease in grip strength between the healthy and the mean affected side

## Appendix 2

**Table:** Missing data

Missing variables	Number of patients (%)
Swelling of distal radius present	1 (0.1)
Swelling of distal ulna present	32 (4.1)
Swelling of anatomical snuffbox	2 (0.3)
Visible deformation	0
Bone tenderness distal radius	2 (0.3)
Bone tenderness distal ulna	3 (0.4)
Bone tenderness anatomical snuffbox	3 (0.4)
Dorsiflexion painful	3 (0.4)
Palmar flexion painful	4 (0.5)
Supination painful	3 (0.4)
Pronation painful	3 (0.4)
Ulnar deviation painful	4 (0.5)
Radial deviation painful	5 (0.6)
Radioulnar ballottement test painful	25 (3.2)
Axial compression of forearm	25 (3.2)
Prehensile grip strength	98 (12.5)

## Appendix 3

**Table:** Interaction of the variables

	Swelling of distal radius	Swelling of distal ulna	Swelling of anatomical snuffbox	Deformation	Bone tenderness distal radius	Bone tenderness distal ulna	Bone tenderness anatomical snuffbox	Dorsiflexion	Palmar flexion	Supination	Pronation	Ulnar deviation	Radial deviation	Radio ulnar ballottement test
Swelling of distal ulna	141													
Swelling of anatomical snuffbox	48	22												
Deformation	50	35	11											
Bone tenderness distal radius	342	146	56	55										
Bone tenderness														
distal ulna	175	147	32	43	346									
Bone tenderness anatomical snuffbox	90	33	59	15	178	102								
Dorsiflexion	319	162	65	55	561	351	193							
Palmar flexion	267	137	60	48	487	307	176	531						
Supination	274	141	51	55	489	306	154	490	439					
Pronation	258	131	47	49	464	291	148	465	426	489				
Ulnar deviation	295	144	54	52	453	311	167	485	451	427	416			
Radial deviation	294	141	61	54	510	301	182	524	479	450	433	471		
Radio ulnar ballottement test	263	135	54	49	464	295	148	446	400	391	377	383	405	
Axial compression of forearm	253	121	50	48	450	277	161	442	405	372	364	387	417	369

## Appendix 4

Radiographs of patients with a potentially missed fracture if applying the Amsterdam Pediatric Wrist Rules in the external validation cohort

Patient 1: A 15-year-old boy with a buckle fracture of the distal radius

Patient 2: A 10-year-old boy with a buckle fracture of the distal radius

Patient 3: A 12-year-old boy with a subtle buckle fracture of the distal radius

Patient 4: A 10-year-old boy with a buckle fracture of the distal radius

Patient 5: An 11-year-old with a fracture of the distal radius with buckle component dorsal

Patient 6: A 10-year-old boy with a buckle fracture of the distal radius

Patient 7: A 9-year-old boy with a buckle fracture of the distal radius
